# Research Progress on the Insecticidal and Antibacterial Properties and Planting Applications of the Functional Plant *Cnidium monnieri* in China

**DOI:** 10.3390/plants15020281

**Published:** 2026-01-17

**Authors:** Shulian Shan, Qiantong Wei, Chongyi Liu, Sirui Zhao, Feng Ge, Hongying Cui, Fajun Chen

**Affiliations:** 1 State Key Laboratory of Agricultural and Forestry Biosecurity (Nanjing), Department of Entomology, College of Plant Protection, Nanjing Agricultural University, Nanjing 210095, China; 2024802146@stu.njau.edu.cn (S.S.); 2023802144@stu.njau.edu.cn (Q.W.); liuchongyi@stu.njau.edu.cn (C.L.); 20220303002@stu.sxau.edu.cn (S.Z.); 2 Shandong Key Laboratory for Green Prevention and Control of Agricultural Pests, Institute of Plant Protection, Shandong Academy of Agricultural Sciences, Jinan 250100, China; gef@ioz.ac.cn

**Keywords:** functional plant, *Cnidium monnieri*, active compounds, insecticidal and antibacterial properties, ecological control

## Abstract

*Cnidium monnieri* (L.) Cusson is a species of Umbelliferae plants, and it is one of China’s traditional medicinal herbs, widely distributed in China owing to its strong adaptability in fields. In this article, the research progress on the taxonomy, distribution, cultivation techniques, active components, analysis methods, antibacterial and insecticidal properties, and ecological applications of *C. monnieri* was reviewed. The main active components in *C. monnieri* are coumarins (mainly osthole) and volatile compounds, exhibiting multiple pharmacological effects, e.g., anti-inflammatory, antibacterial, antioxidant, anti-tumor, and immune-regulating effects. Some modern analytical techniques (e.g., HPLC, GC-MS, and UPLC-QTOF-MS) have enabled more precise detection and quality control of these chemical components in *C. monnieri*. The specific active constituents in *C. monnieri* (e.g., coumarins and volatile components) exhibit significant inhibitory effects against various pathogenic fungi and insect pests. Simultaneously, the resources provided during its flowering stage (e.g., pollen and nectar) and the specific volatiles released can repel herbivorous insect pests while attracting natural enemies, such as ladybugs, lacewings, and hoverflies, thereby enhancing ecological control of insect pests in farmland through a “push–pull” strategy. Additionally, *C. monnieri* has the ability to accumulate heavy metals, e.g., Zn and Cu, indicating its potential value for ecological restoration in agroecosystems. Overall, *C. monnieri* has medicinal, ecological, and economic value. Future research should focus on regulating active-component synthesis, improving our understanding of ecological mechanisms, and developing standardized cultivation systems to enhance the applications of *C. monnieri* in modernized traditional Chinese medicine and green agriculture production.

## 1. Introduction

Functional plants that provide both medicinal value and ecological services are attracting increasing attention in the context of sustainable agriculture and green pest management. *Cnidium monnieri* (L.) Cusson is a commonly used traditional Chinese medicinal herb and has long been valued for its pharmacological properties, which are mainly attributed to coumarin compounds such as osthole [1,2,3].

Beyond its medicinal applications, recent studies have revealed that *C. monnieri* also plays multifunctional roles in agroecosystems. Its coumarins and volatile components exhibit repellent or inhibitory effects against farmland insect pests, while simultaneously attracting natural enemies such as ladybugs, lacewings, and hoverflies, thereby contributing to biological control and ecological regulation in agricultural systems [4,5]. In addition, *C. monnieri* has been reported to possess certain biotransport and enrichment capacities for heavy metals in ecologically restored environments, suggesting further ecological value beyond pest management [6].

Although research on *C. monnieri* has expanded from traditional pharmacological studies to research on its cultivation, phytochemistry, and ecological functions, current knowledge remains fragmented across disciplines. A comprehensive synthesis integrating its chemical composition, biological activities, and ecological roles is still lacking. Therefore, this review aims to summarize and integrate existing studies, which were searched on Web of Science, Elsevier ScienceDirect, Wiley Online Library, Springerlink, CNKI, WanFang Data, “https://www.baidu.com/”, etc., based on the active components, biological properties, and ecological functions of *C. monnieri*, with particular emphasis on its potential application as a multifunctional plant in ecological and sustainable agricultural systems [7].

## 2. Species Classification and Distribution of *C. monnieri*

*C. monnieri* is a representative species of the genus *Cnidium* within the subfamily Apioideae of the family Apiaceae, and it is phylogenetically related to genera such as *Angelica* and *Apium* [8,9]. The genus *Cnidium* has only a few species worldwide, mainly distributed in Eurasia, and China is one of its important distribution centers. In recent years, with the development of molecular systematics, some scholars have used the sequences from ribosomal DNA internal transcribed spacers and chloroplast genes to confirm its stable taxonomic status and reveal its species-specific characteristics among some morphologically similar species [10].

**Figure 1:**
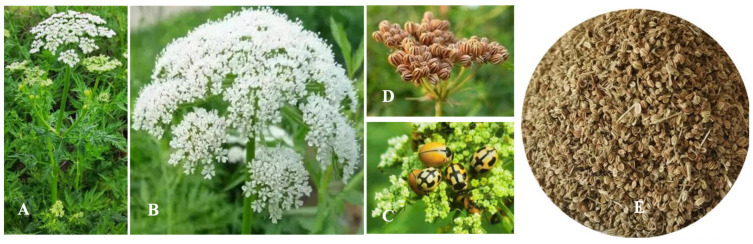
The *Cnidium monnieri* (L.) Cusson plant (**A**); compound umbels with numerous flowers; (**B**) flowers that have attracted ladybugs (**C**); mature flowers (**D**); and harvested seeds (**E**) (note: plant height is 30–80 cm; the diameter of compound umbels is 2.0–3.0 cm; the seed length is 1.0–1.5 mm; and the width is 0.5–0.8 mm).

Morphologically, *C. monnieri* is a herbaceous plant characterized by pinnately divided leaves, compound umbel inflorescences with white flowers, and small schizocarps, which are typical diagnostic features within the Apiaceae family [1,11,12]. The *C. monnieri* plant and its compound umbels with numerous flowers for attracting ladybugs, and the mature flowers and seeds of *C. monnieri*, are shown in Figure 1. The growth and development process of *C. monnieri* includes eight stages, including germination, seedling, tillering, jointing, bud stage, flowering, fruiting, and maturation [13].

Based on its morphological and chemical characteristics, *C. monnieri* can be classified into three types. The first type is characterized by smaller fruits with angular furanocoumarins as main components while lacking osthole or linear furanocoumarins, and it is distributed in the provinces of Heilongjiang, Inner Mongolia, and Northern Liaoning; the second type is characterized by larger fruits with osthole and linear furanocoumarins as main components while lacking angular furanocoumarins, and it is mainly distributed in the provinces of Fujian, Zhejiang, and Jiangsu; the third type is characterized by an intermediate fruit size between that of the first two types, contains osthole, linear furanocoumarins and angular furanocoumarins, and is distributed in the provinces of Shaanxi, Hebei, and Henan [14]. *C. monnieri* is the primary source of the Chinese medicinal material “Shechuangzi”. Although the cultivated and wild resources of *C. monnieri* are currently relatively abundant, its wild populations face threats of decline due to environmental changes and over-harvesting, just like many other medicinal herbs [15]. Therefore, the standardized cultivation of *C. monnieri* should be widely promoted in order to enhance its application potential, which is urgent for achieving its sustainable utilization. Overall, these three types exhibit clear regional differentiation in both morphological traits and chemical composition. Northern populations are more frequently associated with angular furanocoumarins and reduced osthole content, whereas southeastern populations tend to accumulate higher levels of osthole and linear furanocoumarins. The intermediate type displays mixed chemical profiles, suggesting a potential transitional pattern among regional populations [14,16,17]. However, direct quantitative comparison between types remains limited due to differences in sampling strategies, cultivation status, and analytical methods reported in the literature [17,18].

## 3. Cultivation Techniques and Field Management of *C. monnieri*

*C. monnieri* can reproduce through natural seed dispersal and is characterized by strong adaptability to a wide range of environmental conditions. It does not require vernalization and can be cultivated under diverse light, soil, and moisture regimes, indicating its suitability for large-scale and flexible cultivation systems. The species is considered day-neutral, with growth duration varying across geographic regions, and is commonly found in open and well-drained habitats such as field margins, river valleys, grasslands, and hillsides [19,20].

In agricultural practice, *C. monnieri* is generally cultivated on well-drained sandy or loamy soils following conventional land preparation. Standard field management includes appropriate irrigation during early growth stages, basic fertilization to support vegetative and reproductive development, and regular weeding to reduce competition. These practices are consistent with the low nutrient demand and stress tolerance reported for this species, further supporting its potential for integration into ecological and low-input agricultural systems [19,21].

During the growing season, *C. monnieri* may be affected by common diseases and insect pests, such as powdery mildew, spider mites, and aphids. Integrated management strategies combining field sanitation, habitat management, and biological control are commonly recommended, while chemical control is applied only when necessary. Notably, plant-derived compounds from *C. monnieri* itself have been reported to contribute to pest suppression, highlighting its dual role as both a cultivated species and a functional plant in agroecosystems [1,19,21].

## 4. Antibacterial and Insecticidal Properties of *C. monnieri* and Its Ecological Functions in Natural-Enemy Conservation

In recent years, with the rapid development of green and ecological agriculture, research focus has shifted toward the ecological service functions of *C. monnieri* in controlling insect pests and diseases in farmland ecosystems. These functions include antibacterial and insecticidal activities, interference with insect pest behavior, and enhancement of biological control efficacy by providing food resources and habitats for natural enemies. Numerous studies have shown that *C. monnieri* exhibits antibacterial activity against plant pathogenic bacteria, which can inhibit or repel agricultural insect pests. In addition, as a flowering plant with compound umbels, its long flowering period, diverse nectar, and specific volatile compounds can attract and sustain population abundances of natural enemies, e.g., parasitic wasps and hoverflies, predacious lacewings, and ladybugs. For instance, many studies have promoted *C. monnieri* as a “functional plant” for ecological pest management in wheat fields and other crops [15,22,23,24,25,26].

### 4.1. Antibacterial Activity of C. monnieri

Osthole and linear furanocoumarins are among the active components of *C. monnieri* with antifungal activity [27]. The 95% ethanol crude extract from *C. monnieri* showed strong inhibitory activity against the mycelial growth of *Peronophthora litchii*, with an inhibition rate of 53.79% at 5 mg/mL (*p* < 0.05) [28]. Liu et al. (2023) found that the 95% ethanol extract from *C. monnieri* exhibited antibacterial activity against *Botrytis cinerea* with an inhibition rate of 31%, showing no significant difference compared with the inhibition rate of azoxystrobin (49.3%; *p* > 0.05) [29]. Research on the inhibitory effect of different osthole concentrations on *B. cinerea* indicated that the strongest inhibition occurred at a concentration of 5 mg/mL, with an inhibition rate of 93.15% [29]. Shi et al. (2004) discovered that osthole treatment caused hyphal breakage in *Fusarium graminearum*, and osthole at 50 μg/mL significantly inhibited its spore germination (*p* < 0.05); after treatment with 100 μg/mL osthole for 24 h, massive hyphal breakage occurred, soluble protein content increased, glucose content showed a “V”-shaped change, and chitinase activity and chitin content increased [30]. Furthermore, Zheng et al. (2021) reported that osthole significantly inhibited various plant pathogenic fungi (e.g., *Fusarium moniliforme*, *Thanatephorus cucumeris*; *p* < 0.05), causing disordered fungal structure and damage to organelles [31]. Hu et al. (2023) further indicated that osthole disrupted the integrity of the cell wall and cell membrane in fungi such as *Fusarium oxysporum* [32]. Comparing the antibacterial activity of *C. monnieri* extracts with different solvents revealed that the inhibition rate against mycelial growth of various plant pathogenic fungi exceeded 65%, demonstrating its broad-spectrum antibacterial activity [28,31,33]. Taken together, current studies indicate that the antibacterial activity of *C. monnieri*, particularly its major coumarin component osthole, is mainly associated with structural and functional disruption of fungal cells. Microscopic observations have shown that osthole treatment leads to hyphal deformation, breakage, and collapse, accompanied by severe damage to cell walls and membranes [30,31,32]. Biochemical analyses further revealed increased membrane permeability, leakage of intracellular proteins, and disturbances in carbohydrate metabolism, as reflected by abnormal changes in soluble protein, glucose content, and chitinase activity [30].

### 4.2. Insecticidal Activity of C. monnieri

The main active component osthole and its derivatives in *C. monnieri* exhibit insecticidal, antifeedant, and developmental inhibitory activities in in vitro and laboratory bioassays [4,34]. As a botanical insecticide, osthole primarily acts through contact toxicity, and stomach poison activity as a secondary mode. The insecticidal solution is absorbed through the insect’s integument to affect the nervous system, and ultimately leads to death due to exhaustion [4,35]. Osthole shows good contact toxicity and antifeedant effect against agricultural pests, e.g., spider mites and peach-potato aphids. For example, Dong et al. (2023) found that osthole had toxic effects on the first-instar nymphs and adults of spider mites and peach-potato aphids through leaf-contact and choice experiments, exhibiting a dose-dependent effect [4]. Simultaneously, it significantly prolonged the developmental period of both insect pests and reduced their reproductive capacity (*p* < 0.05) [4].

In addition, structurally modified derivatives of osthole have also demonstrated superior activity against insect pests. Li et al. (2021) esterified the lactone ring of osthole, obtaining several ester derivatives, some of which showed 1.6–1.8 times higher insecticidal activity against *Mythimna separata* than that of osthole [36]. Other studies reported the synthesis of oxime ester derivatives from osthole, which induced a higher mortality rate in an inhibition test against certain important insect pests [34]. In summary, osthole and its derivatives from *C. monnieri* not only exhibit inherent insecticidal and behavioral-interference activities, but also show significantly enhanced efficacy after necessary molecular structural modifications (*p* < 0.05). This provides a theoretical basis and research direction for application of *C. monnieri* or its extracts in botanical pesticides. From a mechanistic perspective, the insecticidal activity of osthole and its derivatives appears to involve multiple physiological and behavioral targets. Contact-toxicity and antifeedant assays indicate that osthole can penetrate the insect cuticle and interfere with neural signal transmission, leading to abnormal behavior, reduced feeding, and eventual mortality [4,34,35].

### 4.3. Ecological Function of C. monnieri in Natural-Enemy Conservation

As a functional companion plant, *C. monnieri* plays an important role in natural-enemy conservation by enhancing the abundance, diversity, and persistence of beneficial arthropods in agricultural ecosystems. Field studies have demonstrated that the introduction of *C. monnieri* into cropping systems can significantly increase populations of key natural enemies (*p* < 0.05), including lacewings, hoverflies, ladybugs, and parasitoid wasps, thereby strengthening the biological control potential against insect pests [5,15,37].

From a mechanistic perspective, the ecological functions of *C. monnieri* in natural-enemy conservation can be attributed to multiple complementary factors. *C. monnieri* emits specific volatile organic compounds, such as o-diethylbenzene and p-diethylbenzene, which have strong attractive effects on predatory insects, particularly ladybugs. These volatiles facilitate the recruitment and spatial redistribution of natural enemies from *C. monnieri* stands into adjacent crop fields, consequently enhancing pest suppression efficiency at the field scale [5,15,26]. The characteristic umbel inflorescence and complex branching architecture of *C. monnieri* provide structurally diverse microhabitats that support sheltering, mating, and oviposition of natural enemies. Such habitat complexity increases niche availability and promotes the stability of natural-enemy communities, especially under fluctuating environmental conditions. In addition, *C. monnieri* can harbor non-pest aphid species (e.g., *Semiaphis heraclei*), which serve as alternative prey and enable the maintenance of natural-enemy populations during periods of low pest abundance in crop fields. This function effectively establishes *C. monnieri* as an ecological bridge linking successive cropping cycles.

Pollen and nectar resources supplied by *C. monnieri* act as supplementary food sources for adult natural enemies, supporting their survival, fecundity, and multigenerational persistence. The continuous availability of these nutritional resources contributes to the long-term conservation of natural-enemy populations and enhances their responsiveness to pest outbreaks [5,15,25,38]. It should be noted that interactions among natural enemies, including resource competition and intraguild predation, may occur within *C. monnieri*-associated communities. However, empirical evidence suggests that the net effect of *C. monnieri* incorporation is a positive enhancement of biological control services. The preferential migration of natural enemies toward pest-infested crop fields, driven by higher prey density, further amplifies pest suppression and reinforces the ecological control function of *C. monnieri* in agroecosystems [15,38,39].

## 5. Types and Functions of Active Components in *C. monnieri*

### 5.1. Coumarin Compounds and Their Functions

The main active components in *C. monnieri* are coumarin compounds, primarily osthole, which constitute the principal material basis for its pharmacological efficacy. Coumarins are derivatives of 1,2-benzopyrone, encompassing a class of natural products with diverse molecular structures. Modern phytochemical studies show that *C. monnieri* exhibits broad pharmacological activities and contains various coumarin compounds, including osthole, isopimpinellin, psoralen, isopsoralen, imperatorin, etc. [1,12,16,40,41]. The major coumarin compounds identified in *C. monnieri*, together with their reported biological activities and putative roles, are summarized in Table 1. To date, 48 coumarin compounds have been isolated and identified from *C. monnieri*. Based on their structural differences, they can be classified into three categories, i.e., simple coumarins, linear furanocoumarins, and angular furanocoumarins [17].

Simple coumarin compounds have a core structure consisting of a benzene ring fused with a pyrone ring. Among them, osthole is the representative of simple coumarins, the primary active component of *C. monnieri*, with a wide range of pharmacological activities [42]. As a coumarin compound, osthole possesses the core coumarin structure along with an isopentenyl side chain, and isoprenoid compounds play important roles as phytoalexins in disease resistance [2,16,55]. Linear furanocoumarins are a class of coumarins in which a furan ring is linearly fused to the coumarin core structure, typically with substituents located at the 5 and 8 positions. Representative compounds include osthole, xanthotoxin, xanthotoxol, imperatorin, bergapten, isoimperatorin, isopimpinellin, etc. [41,42,43,45]. Angular furanocoumarins refer to coumarins where the α-pyrone ring, benzene ring, furan ring, or pyran ring forms an angular arrangement, with substituents typically on the furan ring. Angular furanocoumarins with a double bond at the 8 position include angelicin and 2’-acetylangelicin. In contrast, angular furanocoumarins without a double bond at the 8 position include columbianetin, cnidiadin, and columbianadin, and the angular furanocoumarins with substituents at the 8 and 9 positions include archangelicin, edultin, cniforin, etc. [41,42,45]. Structural differences among simple coumarins, linear furanocoumarins, and angular furanocoumarins are closely associated with their biological activities. Variations in prenyl side chains, methoxy substitutions, and ring fusion patterns influence physicochemical properties such as lipophilicity, membrane permeability, and photoreactivity, thereby affecting antimicrobial, insecticidal, and photobiological activities [2,41,42,45]. These structure–activity relationships provide a chemical basis for the diverse bioactivities reported for *C. monnieri* constituents. From a biosynthetic perspective, the coumarins and furanocoumarins identified in *C. monnieri* are generally derived from the phenylpropanoid pathway [2,16]. Subsequent enzymatic modifications, including hydroxylation, O-methylation, and prenylation, contribute to the structural diversification of simple coumarins as well as linear and angular furanocoumarins [41,42]. However, the specific biosynthetic genes and regulatory networks responsible for these processes in *C. monnieri* remain poorly characterized, and current knowledge is largely inferred from studies on other Apiaceae species [56,57].

**Figure 2:**
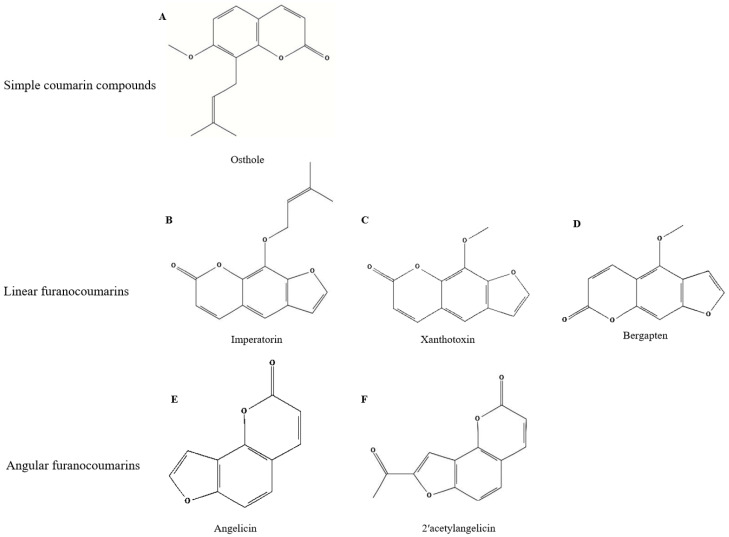
Representative chemical structures of major coumarin compounds from *C. monnieri*. (**A**) Simple coumarins, represented by osthole; (**B**–**D**) linear furanocoumarins, represented by imperatorin, xanthotoxin, and bergapten; (**E**,**F**) angular furanocoumarins, represented by angelicin and 2’-acetylangelicin. The common coumarin scaffold and different furan ring orientations (linear vs. angular) are illustrated to highlight structural diversity among the major bioactive compounds.

Coumarin compounds are the core active substances of *C. monnieri*, with a pharmacodynamic profile primarily including anti-inflammatory, antimicrobial, antipruritic and skin barrier protection; bone metabolism regulation; anti-tumor activity; and smooth muscle regulation. Many studies have shown that coumarin compounds can downregulate the expression of pro-inflammatory factors by inhibiting inflammatory signaling pathways (e.g., NF-κB/MAPK), thereby alleviating inflammation induced by bacteria, fungi, and allergic reactions [1,2,16]. Furanocoumarins are widely used to treat vitiligo and psoriasis due to their photosensitizing properties [44]. Some coumarin substances with hydroxyl and methoxy groups also have antioxidant effects and are often used in cosmetics production [58]. The representative molecular structures of major coumarin compounds in *C. monnieri* are presented in Figure 2.

### 5.2. Volatile Components and Their Olfactory Functional Effects in C. monnieri

In addition to coumarins, *C. monnieri* contains fatty acids (e.g., oleic acid and linoleic acid) and a variety of volatile oils. While these fatty acids are not volatile compounds, they act as precursors for the formation of certain volatile constituents involved in aroma formation and biological activity [51]. The main components of volatile components in *C. monnieri* plants include β-eudesmol, bornyl acetate, p-cymene, limonene, linalool, caryophyllene, and γ-terpinene, and these substances collectively determine the pungent aroma and characteristic fragrance profile of the medicinal material [1,3,7]. Using gas chromatography–mass spectrometry (GC-MS) to analyze the chemical composition of volatile components in *C. monnieri* plants, 50 components were identified, accounting for 86% of the total volatile composition; among them, the compound with the highest relative content was trans-calamenene, followed by selinene [49]. In the volatile components of *C. monnieri*, the relative content of sesquiterpene components is over 85%, esters over 7%, and terpene alcohols over 2% [7]. Xiang et al. (1999) were the first to isolate and identify β-eudesmol and bornyl acetate from *C. monnieri*. Additionally, the volatile components of *C. monnieri* also contains small amounts of ketones, ethers, aldehydes, and benzene derivatives [46,49]. In addition, Zhu et al. (2008) analyzed the volatile components of *C. monnieri* collected from the provinces of Anhui and Henan; there were 36 components identified in Anhui samples, while 45 components were identified in Henan samples, with only 28 common components with significant differences in relative content (*p* < 0.05) [18].

The volatile components of *C. monnieri* are not only pharmacologically active, but also play a dual role in natural ecosystems, including plant defense and signaling. As a traditional medicinal plant, *C. monnieri* is primarily used for insecticidal and antipruritic purposes, and modern research suggests that the material basis is the volatile oil components, providing new guidance for traditional usage patterns [43]. Beyond pharmacological effects, the volatile components of *C. monnieri* also have important ecological functions, and its main components are mostly bioactive terpenoids, which can act as chemical signaling molecules in plant defense systems. When subjected to pest damage or mechanical injury, they are rapidly released, facilitating inter-plant information exchange through odor, thereby inducing defense responses in neighboring plants or directly repelling phytophagous insects [47]. Some monoterpenes, e.g., limonene, p-cymene, and linalool, have been proven to exhibit significant repellent activity against insect pests, capable of interfering with the chemotactic behavior of phytophagous insects, and these compounds can attract certain natural enemies, helping to establish an ecological balance among host plants, insect pests, and natural enemies [48].

### 5.3. Other Active Components in C. monnieri

In addition to the primary active substances (e.g., coumarins and volatile components), *C. monnieri* contains various other bioactive components, including flavonoids, fatty acids, polysaccharides, sterols, and organic acids; these substances collectively constitute the diversified basis for the pharmacological and ecological functions of *C. monnieri* [1,16,42,43]. Although these active components are present in relatively low amounts, they play important complementary and synergistic roles in pharmacological and ecological functions of *C. monnieri*. Duan et al. (2015) isolated 10 chromone compounds from a 75% ethanol extract from *C. monnieri* plants, identified as cnidimoside A, cnidimol B, peucedanocoumarin, 7-O-β-glucosylscoparone, cimifugin, and 5-hydroxychromone-7-O-β-glucoside [50]. *C. monnieri* also contains some steroidal substances, including campesterol, stigmasterol, and γ-sitosterol, and possess certain medicinal value [53]. Meanwhile, relatively high contents of oleic acid (28.85%) and linoleic acid (10.95%) have been detected in *C. monnieri*, and these two unsaturated fatty acids not only participate in maintaining cell membrane structure and signal transduction, but also have anti-inflammatory and cardiovascular protective effects [51,52]. In addition, small amounts of steroidal compounds (e.g., β-sitosterol, stigmasterol) and triterpenoids (e.g., oleanolic acid, ursolic acid) have been identified from *C. monnieri*, and these components have been confirmed to have lipid-lowering, anti-inflammatory, and anti-tumor effects [51,52].

The types and functions of active components in *C. monnieri* are summarized in Table 1. Overall, these compounds participate in antioxidant, immune regulation, metabolic balance, and tissue protection processes through multiple pathways. Future research should systematically analyze the metabolic networks of these active components and their interactions with major active groups using multi-omics technologies, providing a deeper scientific basis for fully elucidating the pharmacodynamic material basis of *C. monnieri*.

## 6. Identification and Analytical Methods for Bioactive Compounds in *C. monnieri*

The bioactive constituents of *C. monnieri* exhibit substantial diversity in polarity, volatility, photostability, and abundance, which poses significant challenges for comprehensive chemical characterization [1,46]. Although a wide range of analytical techniques have been applied (Table 2), the suitability and limitations of each method vary depending on analytical objectives, such as qualitative identification, quantitative determination, or holistic metabolic profiling. Therefore, this section emphasizes a comparative evaluation of commonly used analytical strategies and their applicability to different research purposes in *C. monnieri* studies. In addition, several emerging techniques (e.g., supercritical fluid extraction and capillary electrophoresis) have been explored as complementary tools for sample preparation and quality assessment, although their use remains more application-specific compared with mainstream LC/LC-MS workflows [59,60,61].

Different analytical techniques are suited to different research objectives. Conventional HPLC and GC methods offer a favorable balance between analytical performance, operational cost, and accessibility, making them suitable for routine quality control and large-scale sample analysis of major coumarins and volatile components in *C. monnieri* [17,22,51,62,63,70]. In contrast, advanced techniques such as UPLC-QTOF-MS or metabolomics-based approaches provide higher sensitivity and broader metabolite coverage, enabling comprehensive profiling and quality differentiation, but they require greater financial investment, technical expertise, and data-processing capacity [18,56,57,67,68,69,71]. Therefore, the choice of analytical technique should be aligned with the specific analytical purpose and practical feasibility, as suggested by previous studies [17,67,69].

### 6.1. Chromatographic Analysis Methods

Chromatographic techniques constitute the core tools for separating and quantifying chemical constituents in *C. monnieri*. However, their analytical performance differs markedly across compound classes. Early approaches such as thin-layer chromatography (TLC) and gas chromatography (GC) remain useful for rapid qualitative screening and volatile component analysis, respectively, but are limited by low sensitivity and applicability mainly to thermally stable compounds [22,51,70]. Consequently, these techniques are increasingly inadequate for comprehensive profiling of non-volatile coumarins and trace constituents.

High-performance liquid chromatography (HPLC) has therefore become the dominant method for routine quality control of *C. monnieri*, owing to its high separation efficiency, strong sensitivity, and reliable quantification of major coumarins [17,22,62,63]. Nevertheless, conventional HPLC methods often fail to detect low-abundance components and provide limited structural information without spectrometric coupling. The integration of HPLC with mass spectrometry (LC-MS) significantly enhances analytical depth by enabling simultaneous separation, molecular-weight determination, and tentative structural annotation, thereby extending applicability from targeted analysis toward broader compositional characterization [17,18,28,72].

### 6.2. Spectroscopic and Spectrometric Analysis Methods

Spectroscopic techniques primarily serve as complementary tools for structural elucidation rather than standalone analytical platforms for complex extracts of *C. monnieri*. Ultraviolet–visible (UV-Vis) spectroscopy and Fourier transform infrared (FTIR) spectroscopy provide rapid information on functional groups and characteristic absorption patterns, but their limited specificity restricts their application in multi-component systems [23,64,65,73,74]. Nuclear magnetic resonance (NMR) spectroscopy offers definitive structural confirmation and purity assessment of isolated compounds, yet requires relatively large sample quantities and is unsuitable for high-throughput analysis [58,65,66].

Mass spectrometry (MS), particularly when coupled with chromatographic separation, overcomes many of these limitations by providing high sensitivity and broad molecular coverage. Liquid chromatography–mass spectrometry (LC-MS) is widely applied for rapid screening, molecular-mass determination, and metabolite profiling of *C. monnieri* extracts, making it an indispensable technique for bridging targeted compound identification and system-level chemical analysis [71].

### 6.3. Metabolomics Analysis

Given the chemical complexity and multi-component nature of *C. monnieri*, metabolomics has emerged as an effective strategy to overcome the limitations of single-compound analytical approaches. In contrast to traditional targeted analyses focusing on a limited number of markers, metabolomics enables simultaneous detection of a broad spectrum of metabolites and reveals compositional variation at a systems level [69].

UPLC–high-resolution mass spectrometry (HRMS)-based metabolomics, combined with multivariate statistical analysis, allows comprehensive characterization of secondary metabolite profiles, quality differentiation, and identification of key bioactive components associated with efficacy [56,57,67,68]. However, these approaches also face challenges related to data volume, metabolite annotation accuracy, and methodological standardization. Despite these limitations, metabolomics represents a critical methodological advance by shifting *C. monnieri* research from descriptive chemical analysis toward integrative, mechanism-oriented investigation [56,69].

## 7. Other Applications of *C. monnieri*

### 7.1. Ecological Restoration Value

With the advancement of agricultural green transformation and ecological civilization construction, the role of medicinal and functional plants in modern agriculture and environmental management is gradually shifting from just production objects to ecosystem function regulators [75,76]. Beyond traditional functions, e.g., photosynthesis and material cycling, these plants are increasingly being applied in areas such as soil remediation, microecological regulation, and biodiversity maintenance [77,78]. In farmland ecosystems, plants form complex interaction networks with soil microorganisms and environmental factors. Root exudates regulate plant–soil feedback by influencing microbial communities, while leaf volatiles and litter affect soil physicochemical properties and microbial community structure through their impact on soil microorganisms and environmental factors [79,80]. In heavy-metal-contaminated or degraded soil environments, certain plants with strong environmental adaptability and accumulation capacity can absorb, immobilize, or stabilize pollutant elements through phytoremediation processes, thereby restoring ecological functions while reducing pollution risks [81,82,83,84,85,86].

In recent years, the problem of excessive heavy metals in soil has become increasingly prominent with the accumulation of pesticides, chemical fertilizers, and industrial pollutants in agricultural production. Screening medicinal plants with ecological restoration potential has become an important direction for green remediation [87,88]. *C. monnieri* not only has strong tolerance and a well-developed root system, but also exhibits a certain capacity for the accumulation and translocation of various heavy-metal elements (e.g., Cd, Pb, Cu, Zn) [6,89]. Chen et al. (2021) compared the metal absorption capacities of various herbaceous plants; the results indicated that the translocation factors of *C. monnieri* for Zn and Cu were 0.8–1.57 and 0.67–1.13, respectively, indicating a certain upward-transport ability [6]. Therefore, *C. monnieri* can serve as a candidate herbaceous species for the ecological remediation of heavy-metal-contaminated forest understories, capable of tolerating pollution while accumulating metal elements in its aboveground parts, demonstrating potential for use in both ecological remediation and medicinal application.

### 7.2. Landscape and Ecological Service Value

*C. monnieri* has high potential for use not only in ecological insect pest control and medicinal applications, but also as a product with “added value” in farmland ecosystems. In terms of ecological landscaping, as an Apiaceae plant, its open compound umbel structure and prolonged flowering period provide good nectar and pollen source value, offering nutrients and habitat resources for pollinators and predatory insects, e.g., bees, hoverflies, and parasitic wasps. For ornamental and greening purposes, the slender branches, white inflorescences, and moderate plant size of *C. monnieri* make it suitable as a border plant or as an ornamental flower strip for beautifying agricultural landscapes [5,15,25].

### 7.3. Economic and Nutritional Value

In terms of economic value, due to its traditional medicinal status, *C. monnieri* holds development potential in fields such as traditional Chinese medicine, health products, and skincare products. Literature reports indicate that the active components of *C. monnieri* possess anti-inflammatory, antibacterial, antioxidant, and vasodilation-regulating activities [2,42,43]. Before flowering, the stems and leaves of *C. monnieri* are verdant, lush, and tender in texture, and have a unique aroma. They are rich in nutrients, e.g., vitamins, minerals, and dietary fiber, which are beneficial to human health, and they can be consumed as high-quality wild vegetables, and the leaves and young shoots of *C. monnieri* can also be used in condiments [90]. Although research on *C. monnieri* has been largely conducted in China, the ecological functions and bioactive mechanisms summarized in this review may provide valuable references for the development and application of functional companion plants in diverse agroecosystems worldwide.

In summary, the ecological and economic value of *C. monnieri* is manifested at multiple levels; it can serve as a nectar/pollen source plant to enhance ecological services in farmland, as a landscape plant to improve the esthetic appeal of farmland, and as an economic plant to enhance its potential for use in traditional Chinese medicine and derived products. Future integration of these comprehensive applications of *C. monnieri* within ecological agricultural systems could achieve a “win–win” or even “multi-win” situation in terms of both ecological benefits and economic interest.

Current evidence for the ecological application of *C. monnieri* is primarily derived from short-term or site-specific studies, and long-term field evaluations across different agroecosystems are still insufficient [5,15,25,38]. Moreover, although ecological pest management strategies are often considered environmentally beneficial, comprehensive economic assessments comparing *C. monnieri*-based approaches with conventional chemical control are rarely reported, with most studies focusing on ecological outcomes rather than agronomic or economic performance [15,25].

In comparison with conventional pest control methods, *C. monnieri*-based ecological regulation emphasizes the enhancement of ecosystem services, such as natural-enemy conservation and behavioral interference, rather than immediate pest elimination [5,15,26]. Future research should therefore integrate long-term field trials with economic and agronomic assessments to evaluate the practical feasibility and comparative advantages of this approach across different cropping systems [15,38].

## 8. Future Prospects for Research on *C. monnieri*

*C. monnieri* possesses medicinal, ecological, and economic value, and the research on *C. monnieri* is gradually expanding from traditional pharmacological aspects to molecular mechanisms and ecological functions. The coumarins, volatile constituents, and other secondary metabolites of *C. monnieri* constitute its main active material basis, exhibiting various biological effects, e.g., anti-inflammatory, antibacterial, antioxidant, insecticidal, and repellent effects [2,16]. However, systematic research on the biosynthetic regulatory pathways and action mechanisms of these active components is still lacking, and the pharmacodynamic material basis of *C. monnieri* requires further elucidation. Future studies should integrate metabolomics and molecular biology approaches to thoroughly elucidate the secondary metabolic network and the regulatory mechanisms of the expression of key genes [56,57]. It should be noted that the reported bioactivities and ecological effects of *C. monnieri* vary across studies, which may be attributed to differences in experimental design, test organisms, extraction methods, concentrations, and evaluation criteria. Its strong accumulation and translocation capacity in heavy-metal-contaminated soil also indicates its potential value for ecological restoration [6]. On the other hand, the natural-enemy conservation and insect pest control functions of *C. monnieri* in farmland ecosystems provide important technical support for ecological agriculture [5,15]. Future research could further expand on the standardized cultivation, quality control, and comprehensive utilization of *C. monnieri*, aiming to synergistically enhance its medicinal value and ecological benefits, thereby providing theoretical and technical support for the transformation of this functional plant in Chinese medicinal material production and its application in insect green pest control.

At the mechanistic level, several specific research questions remain unresolved. For example, it remains unknown which key biosynthetic enzymes and regulatory genes control the accumulation of osthole and furanocoumarins in different *C. monnieri* populations [2,16,56], and to what extent genetic variation and environmental factors jointly determine the observed chemical diversity and bioactivity [14,67,69]. Addressing these questions will help establish testable links between genotype, metabolite profiles, and biological functions.

Despite its promising potential, several challenges may limit the practical implementation of *C. monnieri*-based applications. These include chemical composition variability among varieties and regions [14,15], difficulties in standardizing cultivation and processing practices [19,21], uncertainty its regarding long-term field stability [5,15,38], and limited economic and regulatory evaluation [15,25]. Addressing these challenges will be essential for translating experimental findings into reliable agricultural practices.

## Figures and Tables

**Table 1:** Representative active components of *C. monnier* and their biological functions.

Compound Category	Representative Compounds	Putative Roles in Plants	Effects on Other Organisms	References
Coumarin-type compounds	Osthole	Plant chemical defense and stress adaptation, often enriched in reproductive tissues and associated with protection	Anti-inflammatory, antibacterial, antifungal, antitumor, antioxidant; smooth muscle modulation; can downregulate the expression of pro-inflammatory cytokines by inhibiting the NF-κB/MAPK signaling pathways	[1,2,16]
	Isoimperatorin; imperatorin	Plant defense-related secondary metabolites, commonly implicated in protection against pathogens/herbivores	Antibacterial, anti-inflammatory, antioxidant; inhibiting the growth of pathogenic fungi	[42,43]
	Psoralen; isopsoralen	Photoactive furanocoumarins implicated in plant defense	Photosensitizing activity; medical use in skin disorders	[44]
	Xanthotoxin; xanthotoxol	Photoactive furanocoumarins functioning in plant chemical defense	Photosensitizing furanocoumarin with antifungal and antitumor activities	[42,45]
	Oxypeucedanin; columbianetin	Putative roles in plant defense/stress responses as furanocoumarin-related metabolites	Anti-inflammatory and neuroprotective activities	[41,45]
	Angelicin; cniforin B	Putative defensive and signaling-associated roles as angular furanocoumarins in plants	Involved in immune regulation and metabolic homeostasis	[41,42]
Volatile components	β-eudesmol	Volatile-mediated plant defense signaling; deterrence of herbivores	Anti-inflammatory, antibacterial; insect-repellent	[7,46]
	Bornyl acetate	Aroma formation; volatile-mediated signaling associated with plant defense	Has soothing, sedative, and antibacterial effects	[1,46]
	p-cymene; limonene	Herbivore deterrence and volatile signaling in plant defense	Strong repellent activity; interference with insect chemotaxis	[47,48]
	Linalool	Attraction of natural enemies as an indirect defense mechanism and plant-to-plant signaling	Exhibits attractant effects on natural-enemy insects and contributes to the maintenance of ecological balance	[48]
	Caryophyllene; γ-terpinene	Stress- and defense-related volatile signals involved in plant defense responses	Antibacterial and antioxidant activities	[7,49]
Other active components	Flavonoids (e.g., chromones, cnidimosides A/B)	Antioxidative protection, UV/stress tolerance, and defense-associated functions in plants	Antioxidant, anti-inflammatory, and immunomodulatory activities	[42,50]
	Fatty acids (oleic acid, linoleic acid)	Membrane structure and signaling precursors; precursors for oxylipins/volatile formation involved in defense signaling	Anti-inflammatory and cardiovascular protective effects	[51,52]
	Steroidal compounds (β-sitosterol, stigmasterol)	Structural and signaling roles in plants as phytosterols involved in membrane integrity and stress responses	Lipid-lowering, anti-inflammatory, antitumor activities	[7,53]
	Triterpenoids (oleanolic acid, ursolic acid)	Putative protective/defense-associated roles in plants	Antitumor, antioxidant, and tissue-protective activities	[54]
	Polysaccharides and organic acids	Primary metabolism and stress adaptation, with potential contributions to plant resilience and growth–defense balance	Immunomodulatory and antioxidant activities; synergistic effects	[1,16]

**Table 2:** Identification of and methods of analyzing active constituents in *C. monnieri*.

Method Category	Representative Techniques	Analytical Principles /Detection Basis	Advantages	Limitations	Main Applications	References
Chromatographic analysis	TLC, HPLC, GC, GC-MS, LC-MS	Separation and detection are achieved based on differences in compound partitioning; adsorption and molecular recognition between the stationary and mobile phases	High separation efficiency, strong sensitivity, accurate quantification, suitability for multi-component detection	Complex sample pretreatment and high instrument requirements; limited detection of thermally unstable or highly polar compounds	Qualitative and quantitative analysis of major active constituents of *C. monnieri*, including coumarins and volatile oils	[17,22,51,62,63]
Spectroscopic/spectrometric analysis	UV-Vis, FTIR, NMR, MS, HPLC-MS	Based on characteristic molecular absorption, vibrational transitions, nuclear magnetic resonance signals, and ion-fragmentation patterns	Rapid analysis, Low sample consumption, capability to provide structural and functional-group information	The resolution is limited and requires validation in combination with other techniques; signal overlap may occur in certain complex samples	Structural elucidation and functional-group identification of coumarins, phenols, alcohols, and other constituents in *C. monnieri*	[23,64,65,66]
UHPLC-HRMS	UPLC-QTOF-MS, UPLC-Orbitrap-MS	Molecular-mass determination and structural identification of components	Extreme sensitivity and high resolution, suitable for both targeted and untargeted analyses	High instrument cost and complex data processing; specialized software is required for interpretation	Systematic analysis of the complex extracts and metabolite profile of *C. monnieri*	[67,68]
Metabolomics analysis	LC-MS/MS, GC-MS, UPLC-QTOF-MS, chemometrics	Systematic detection of changes in multiple metabolites within biological samples, integration with statistical and pathway analyses, reveals compositional differences	Comprehensive and systematic, capability to reveal metabolic pathways, suitable for quality evaluation and differential analysis	Large data volume and complex data interpretation; requirement for validation using conventional analytical methods	Revealing the metabolic characteristics and quality differences in active constituents in *C. monnieri*	[56,57,69]
Other emerging techniques	Supercritical fluid extraction, capillary electrophoresis	High solvation capacity for supercritical fluids, electric-field-driven separation capability	High extraction efficiency, environmentally friendly and safe; electrophoresis is suitable for the analysis of small molecules and ions	Low degree of method standardization; suitable for non-polar macromolecular compounds	Sample preparation of *C. monnieri* extracts, multicomponent separation	[59,60,61]

## Data Availability

The original contributions presented in this study are included in the article. Further inquiries can be directed to the corresponding author.
